# Does a metoclopramide CRI reduce the risk of regurgitation associated with BOAS surgery in dogs?

**DOI:** 10.18849/ve.v11i2.738

**Published:** 2026-05-18

**Authors:** Neil Fraser+

**Keywords:** STRESS

## Abstract

**PICO question:**

In dogs undergoing brachycephalic airway obstruction syndrome (BOAS) surgery does intraoperative constant rate infusion (CRI) of metoclopramide reduce the risk of regurgitation in the perioperative period?

**Clinical bottom line:**

**Category of research:**

Treatment.

**Number and type of study designs reviewed:**

There were no publications that directly answered the PICO question. Two studies were identified which combined the use of metoclopramide in combination with other measures to reduce perioperative regurgitation.

**Strength of evidence:**

Zero.

**Outcomes reported:**

There is no data that solely assesses the effect of metoclopramide on gastro-oesophageal reflux in brachycephalic patients undergoing airway surgery.

**Conclusion:**

Given the lack of evidence relating to the PICO question, the decision to use metoclopramide is based on personal preference and experience. Other medications and anaesthetic protocols that have been shown to reduce perioperative regurgitation should be considered first.

How to apply this evidence in practice

The application of evidence into practice should take into account multiple factors, not limited to: individual clinical expertise, patient’s circumstances and owners’ values, country, location or clinic where you work, the individual case in front of you, the availability of therapies and resources.

Knowledge Summaries are a resource to help reinforce or inform decision making. They do not override the responsibility or judgement of the practitioner to do what is best for the animal in their care.

## Clinical scenario

A 2-year-old French Bulldog presents to your practice for increased respiratory noise. You assess the dog and diagnose the patient with Grade III Brachycephalic Obstructive Airway Syndrome (BOAS). You have a surgeon at your practice capable of performing airway surgery to alleviate the associated symptoms of BOAS. You have found a high percentage of these patients have experienced marked perioperative regurgitation. You wish to find evidence which assesses the use of metoclopramide in reducing these episodes of regurgitation.

## The evidence

Based on a literature search, there was no evidence identified that directly answered the PICO question. Four papers were identified which were relevant but ultimately did not answer the question directly which is why they have been excluded (Costa et al,. 2020; Favarato et al., 2012; Wilson et al., 2005a and Rovatti et al., 2024).

## Appraisal, application and reflection

Gastro-oesophageal reflux (GOR) and regurgitation are well-recognised conditions in brachycephalic breeds, with up to 84% of patients experiencing some degree of reflux in a 24-hour period (Appelgrein et al., 2022). Brachycephalic dogs undergoing BOAS surgery are also three times as likely to experience peri- or postoperative regurgitation when compared to other canine patients (Fenner et al., 2019). Therefore, it is vital to identify ways to reduce the incidence of regurgitation in these patients and prevent the development of aspirational pneumonia.

Although there is no published research into the effect of metoclopramide on GOR when given as a constant rate infusion (CRI) in patients undergoing BOAS surgery, there is contradictory evidence that when given at high doses it will reduce GOR in anaesthetised canines undergoing surgery. Wilson et al. (2005a) offers weak evidence that metoclopramide will reduce GOR when given at high doses in anaesthetised patients. However, Favarato et al. (2012) did not find a significant decrease in GOR following metoclopramide administration at the same higher dose range. The difference in findings here could be due to the use of morphine as a premedication in Wilson et al. (2005a), but not in Favarato et al. (2012). Morphine has been shown to significantly increase the risk of GOR (Wilson et al., 2005b), and the overall incidence of GOR was higher in both Wilson papers compared to Favarato et al. (2012).

A study assessing the incidence of GOR in brachycephalic dogs undergoing spinal surgery treated with a metoclopramide CRI at 2 mg/kg/day did not find any significant decrease in the treated patients (Rovatti et al., 2024). This was a randomised, blind control trial so it provides high quality evidence despite the relatively small sample size of 43 patients.

Costa et al. (2020) found that when metoclopramide is used as part of a preanaesthetic protocol which also implemented famotidine administration and restriction of opioid usage, there was a significant decrease in the incidence of GOR. This, however, was a crossover trial, which does leave room for bias in the results and cannot directly assess the impact of metoclopramide on GOR.

These papers have not been included in the Knowledge Summary as they do not directly address the PICO question. Rovatti et al. (2024) assessed the regurgitation risk in patients undergoing spinal surgery, whereas Costa et al. (2020) and Favarato et al. (2012) assessed regurgitation risk with a combined protocol including metoclopramide so the results cannot solely be attributed to its use.

In summary, there is weak and conflicting evidence regarding whether metoclopramide will reduce the incidence of gastro-oeseophageal reflux in canine patients undergoing surgery, but crucially there is no published data which directly relates to the PICO question. Therefore, further study would be beneficial in this area.

## Methodology

**Search strategy:** 

Databases searched and dates covered:	CAB Abstracts on OVID Platform 1973 to Week 32 2025PubMed on the NCBI Platform 1920 to Week 32 2025
Search strategy:	CAB Abstracts/PubMed: (dog OR dogs OR canines) AND (brachycephalic OR brachy*) AND (metoclopramide OR metoclop*)
Dates searches performed:	09 August 2025

**Exclusion / Inclusion Criteria:** 

Exclusion:	Articles not relevant to the PICO question. Duplicated articles.
Inclusion:	All articles relevant to the PICO question.

**Search Outcome:** 

Database	Number of results	Excluded – not relevant to PICO question	Total relevant papers
CAB Abstracts	4	4	0
PubMed	4	4	0
Total relevant papers when duplicates removed			0

## ORCiD

Neil Fraser: https://orcid.org/0009-0009-4245-3861

## Conflict of Interest

The authors declare no conflicts of interest.

## Disclaimer

Knowledge Summaries are a peer-reviewed article type which aims to answer a clinical question based on the best available current evidence. It does not override the responsibility of the practitioner. Informed decisions should be made by considering such factors as individual clinical expertise and judgement along with patient’s circumstances and owners’ values. Knowledge Summaries are a resource to help inform and any opinions expressed within the Knowledge Summaries are the author's own and do not necessarily reflect the view of the RCVS Knowledge. Authors are responsible for the accuracy of the content. While the Editor and Publisher believe that all content herein are in accord with current recommendations and practice at the time of publication, they accept no legal responsibility for any errors or omissions, and make no warranty, express or implied, with respect to material contained within. For further information please refer to our Terms of Use.
